# Trunk kinematics and low back pain during pruning among vineyard workers—A field study at the Chateau Larose-Trintaudon

**DOI:** 10.1371/journal.pone.0175126

**Published:** 2017-04-06

**Authors:** Romain Balaguier, Pascal Madeleine, Kévin Rose-Dulcina, Nicolas Vuillerme

**Affiliations:** 1 Univ. Grenoble Alpes, AGEIS, Grenoble, France; 2 Physical Activity and Human Performance group—SMI, Dept. of Health Science and Technology, Aalborg University, Aalborg, Denmark; 3 Institut Universitaire de France, Paris, France; Rush University Medical Center, UNITED STATES

## Abstract

The prevalence of low back disorders is dramatically high in viticulture. Field measurements that objectively quantify work exposure can provide information on the relationship between the adopted trunk postures and low back pain. The purposes of the present study were three-fold (1) to carry out a kinematics analysis of vineyard-workers’ pruning activity by extracting the duration of bending and rotation of the trunk, (2) to question separately the relationship between the duration of forward bending or trunk rotation with low back pain intensity and pressure pain sensitivity and (3) to question the relationship between the combined duration of forward bending and trunk rotation on low back pain intensity and pressure pain sensitivity. Fifteen vineyard-workers were asked to perform pruning activity for 12 minutes with a wireless triaxial accelerometer placed on their trunk. Kinematic analysis of the trunk showed that vineyard-workers spent more than 50% of the time with the trunk flexed greater than 30° and more than 20% with the trunk rotated greater than 10°. These results show that pruning activity lead to the adoption of forward bended and rotated trunk postures that could significantly increase the risk of work related musculoskeletal disorders in the low back. However, this result was mitigated by the observation of an absence of significant association between the duration of forward bending and trunk rotation with low back pain intensity or pressure pain sensitivity. Even if prospective field measurements and studies assessing the effects of low back pain confounders are needed, this field study provides new genuine information on trunk kinematics during pruning activity.

## Introduction

Work related musculoskeletal disorders (WMSDs) affecting the low back are considered in numerous industrialized and developed countries as a major public health problem [1–4]. For instance, Farioli and colleagues [5] have recently reported a 46% one year prevalence for low back pain (LBP) among almost 35 000 European workers. The consequences of LBP include disability, early retirement, healthcare consumption, loss of productivity and sickness absences [6,7]. Among all the working sectors, the highest rate of LBP is commonly observed in agriculture [5]. Thereby, in a recent review on the prevalence of WMSDs among farmers, Osborne and colleagues [8] have reported respectively a 75% lifetime and a 48% one year prevalence of LBP. In France, the viticulture sector, which employs more than 500 000 persons, is the agricultural sector with the highest prevalence of WMSDs in the low back [9,10]. Although the origin of LBP is multifactorial, biomechanical risk factors such as heavy physical workload, repetitive motions, awkward postures—especially excessive forward bending and rotation of the trunk—are known to increase the risk of new and recurrent episodes of LBP [11–17]. Interestingly, the few studies assessing WMSDs risk factors among vineyard-workers have also reported an exposure to these biomechanical risk factors especially during the winter job activities such as fixing and pruning [9,18–21]. In an epidemiological study among almost 4 000 French vineyard-workers, Bernard and colleagues [9] have concluded that the postural constraints during pruning activity could increase the risk of LBP. Meyers and colleagues [18], using an observational checklist, have reported that the risk of LBP was increased during pruning due to frequent trunk flexion up to 90°. However, biomechanical exposure in these afore-mentioned studies have been assessed using self-reported measurements or observational methods which can tend to overestimate the time of exposure to risk factors [22–24]. Kato and colleagues [21] have conducted an experimental study addressing the effects of different pruning trellis systems on the risk of developing WMSDs in the lower back. However, a single field study has to our knowledge assessed trunk postures among vineyard-workers during pruning [25]. At this point, this study presents two major limitations. First, it was focused on the assessment of trunk thigh angle in the sagittal plane, while numerous studies have highlighted the effect of the duration of trunk forward bending and trunk rotation on the risk of LBP [26–28]. Second, it did not assess the association between physical exposure and risk of LBP among vineyard-workers, while numerous studies have highlighted the need to evaluate more precisely this association using objective and quantitative field measurements [16,29,30]. As mentioned in numerous studies [31,32], one valid approach to quantify the risk of LBP among workers is to assess the relationship between duration of forward bending and self-reported LBP intensity, e.g. using numeric pain rating scale (NRS). Such analysis can be complemented by measurements of pressure pain thresholds over the low back. Consequently, assessing pressure pain sensitivity over locations of the low back offers an interesting and reliable [33,34] opportunity to investigate and visualize the associations of trunk forward bending, trunk rotation and pain sensitivity.

The purposes of this field study were three-fold:

(1) to carry out a kinematics analysis of vineyard-workers’ pruning activity by extracting the duration of forward bending and rotation of the trunk, that is two factors that are recognized to predispose to low back disorders [16,26–28,35];

(2) to assess separately the relationship between the duration of forward bending or trunk rotation on LBP intensity and pressure pain sensitivity; and

(3) to question the relationship between the combined duration of trunk forward bending and trunk rotation with LBP intensity and pressure pain sensitivity.

## Material and methods

### Description of pruning activity

**Fig 1:**
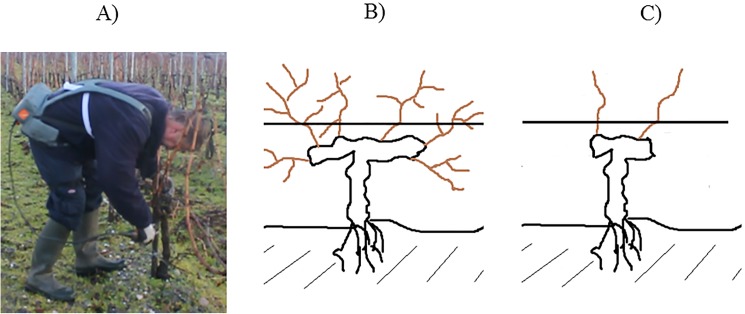
**Common postures adopted by vineyard-worker posture during pruning (A). Cep vine before (B) and after pruning (C)**.

In France, pruning activity generally occurs over 5 months (from November to March). This activity aims at controlling the vine’s development to avoid the production of branches at the expense of grapes. To limit the growth of the vine cep, vineyard workers have to cut precisely some branches, approx. between 25 and 50 cuts per minute [20] with a pruning shear to finally keep 2 main branches that will bear the grapes (Fig 1A, 1B and 1C). At Château Larose-Trintaudon (France), this activity is generally performed both by men and women.

### Participants

Fifteen out of the 24 vineyard-workers employed by the Chateau Larose-Trintaudon (France) volunteered to participate in the study. Table 1 shows the characteristics of these participants. The study was conducted in accordance with the Declaration of Helsinki and approved by the local ethics committee (French society for independent-living technologies and gerontechnology). Written informed consent was obtained from all vineyard-workers included in this study. The participants gave their written informed consent (as outlined in PLOS consent form) to publish these case details. In addition, all the collected data were managed by the MedSafe technology by the IDS Company (Montceau-les-Mines, France). IDS is an approved hosting provider in personal health data by the French Ministry for Social Affairs and Health. Some of the results have been briefly presented during the 6^th^ annual meeting of the Danish Biomechanical Society.

**Table 1:** Characteristics of the vineyard-workers.

Variables	Women (n = 6)	Men (n = 9)
**Age (years)**	48.8 (4.1)	43.0 (7.6)
**Height (cm)**	163.2 (4.8)	171.7 (7.0)
**Body mass (kg)**	68.5 (13.9)	78.7 (14.3)
**BMI (kg/m**^**2**^**)**	25.6 (4.0)	26.5 (3.2)
**Job seniority (years)**	20.5 (3.6)	17.6 (8.0)
**Right-handed (n)**	5	9
**Left-handed (n)**	1	0

Mean (SD)

### Data collection

Data was collected over 8 weeks from January to March 2014. Trunk kinematic was recorded using one wireless inertial measurement unit combining a 3D angular gyroscope, a 3D accelerometer and a 3D magnetometer (I4 motion, Technoconcept, Mane, France; sampling frequency: 100 Hz) and fixed with an adjustable elastic belt to the chest of the participants at the level of the sternum [36]. This location was preferred to the back area often chosen to monitor trunk movement [32,37,38] insofar the vineyard-workers usually carry a harness with a battery placed in this body region. Then, vineyard-workers were asked to perform pruning activity for a period of 12 minutes [25].

### Low back pain intensity and pressure pain sensitivity

A numeric rating scale was used to assess pain intensity of the low back region over the two weeks prior to the data collection. Vineyard-workers were asked to rate their pain intensity using a 0–10 numeric rating scale (0:”No pain”, 10: “Worst imaginable pain”) [25,31] every working day over the 2 weeks prior data collection. The mean of these ratings was used for data analysis enabling to assess the relationship between trunk kinematics and the pain intensity representing a proxy of the pain commonly reported in the low back region by the participants from the Chateau Larose-Trintaudon (France).

**Fig 2:**
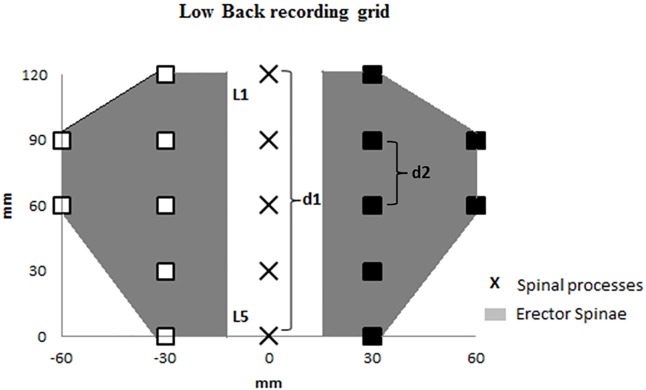
Schematic representation of the low back pressure pain threshold recording grid of the left (blank square) and right (black squares) erector spinae muscles. d1 represents the distance between the first (L1) and the fifth (L5) lumbar vertebrae. d2 equals one fourth of d1.

Pressure pain sensitivity of the lower back region was assessed by measuring PPT over 14 anatomical locations in the low back region (Fig 2) of the vineyard-workers [33,34]. For the analysis, the 7 anatomical locations placed to the left side of the spinal processes have been grouped as P_left_, the 7 anatomical locations placed to the right side of the spinal processes have been grouped as P_right_ and the 14 locations placed to the left of the spinal processes have been grouped as P_all_. For that purpose, a handheld electronic algometer (Somedic, Algometer Type 2, Sollentuna, Sweden) with a 1cm^**2**^ wide rubber tip was used. The examiner measured PPT a constant slope of 30 kPa/s, 3 times on each anatomical location. The mean of 3 PPT measurements of all 14 locations was used for data analysis [33,34,39]. PPT were collected during one session lasting approx. 30 minutes in the 2 weeks prior to the data collection.

### Statistical analyses

**Fig 3:**
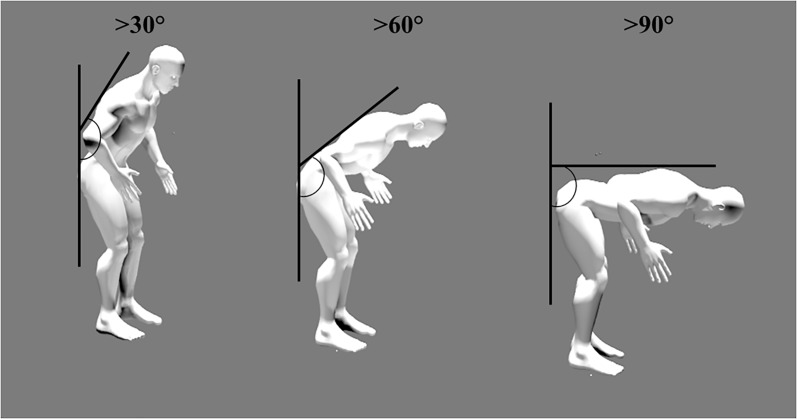
Graphical representation of cut-off angles (i.e. >30°, >60° and >90°) for trunk forward bending.

**Fig 4:**
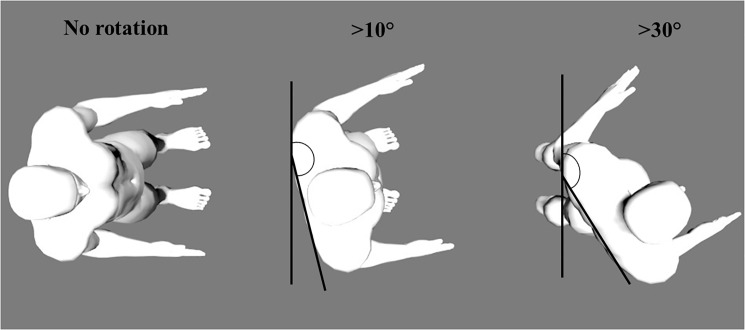
Graphical representation of cut-off angles (i.e. >10° and >30°) for trunk rotation.

Trunk flexion and trunk rotation were categorized from cut-off angles commonly used in the literature. On the one hand, the selected trunk forward bending cut-off angles were the following: <30°, >30°,>60° and >90° [16,26,31,32,40,41] (Fig 3). On the other hand, the selected trunk rotation cut-off angles were the following: <10°, >10° and >30° [27,28] (Fig 4). Percentage of time spent in each cut-off angle was calculated. As data did not follow a normal distribution, Mann-Whitney or Wilcoxon tests were performed to compare the percentage of time spent in each cut-off angle.

Furthermore, Spearman rank correlation coefficient was used to assess the strength and the direction of the association between pressure pain sensitivity or LBP intensity and the time spent in each cut-off angle separately for trunk forward bending and trunk rotation. Then, a sensitivity analysis using a median split to equally separate into 2 groups our sample of vineyard-workers [33,34,42,43] was performed for all cut-off angle to assess whether LBP intensity or pressure pain sensitivity was different between vineyard-workers below or above the median split. Finally, scatter plots were generated to assess the relationship between the combined duration of forward bending and trunk rotation with LBP intensity and pressure pain sensitivity. *P*-values <0.05 were considered statistically significant. All data analyses were performed with R 3.0.1 software (R foundation for Statistical Computing, 2013, Vienna, Austria). Results are presented as median, 25^th^ and 75^th^ percentiles, unless otherwise indicated.

## Results

### Kinematic analysis of the trunk

#### Forward bending of the trunk

**Fig 5:**
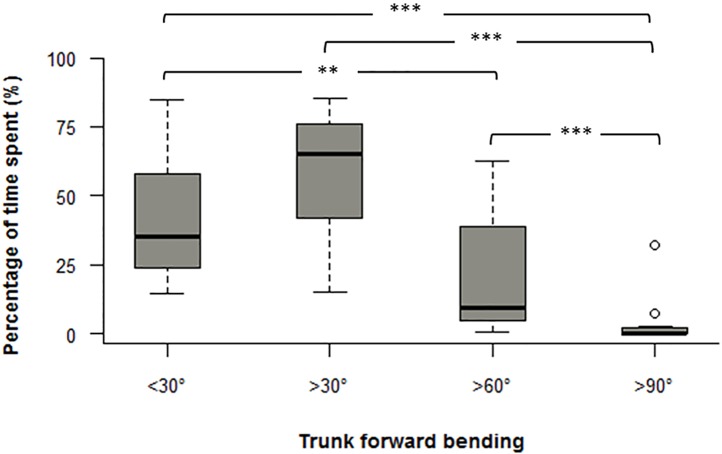
Pruning boxplot of the percentage of time spent at each cut-off angles for trunk forward bending *: *P* < 0.05; **: *P* < 0.01; ***: *P* < 0.001.

Fig 5 shows that more than 50% of time was spent with trunk bended forward >30°. Furthermore, vineyard-workers spent significantly more time with the trunk bended forward >30° compared to <30° (*P*<0.05).

#### Rotation of the trunk

**Fig 6:**
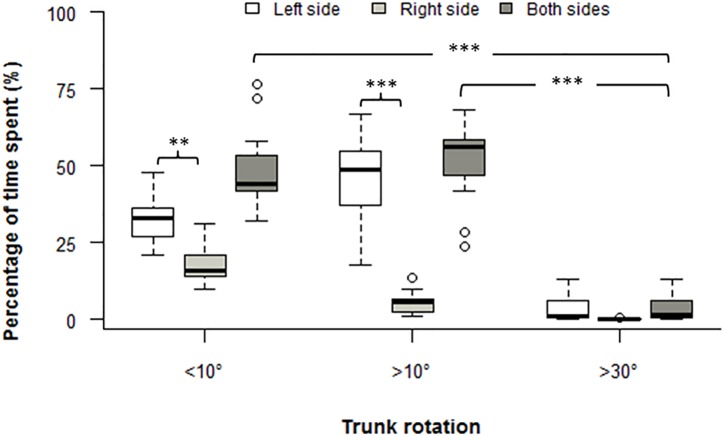
Pruning boxplot of the percentage of time spent at each cut-off angles for trunk rotation *: *P* < 0.05; **: *P* < 0.01; ***: *P* < 0.001.

Fig 6 shows that approx. 50% of the time was spent with the trunk rotated >10°. Furthermore, vineyard-workers spent significantly more time with the trunk rotated on the left side compared with the right side for all the cut-off angles excepted for >30° (*P*<0.05).

#### Relationship between duration of forward bending or rotation of the trunk with LBP intensity and pressure pain sensitivity

No significant correlation (Spearman rank coefficient) between the duration of forward bending of the trunk and LBP intensity or PPT was found significant. The Spearman rank correlation coefficients ranged from -0.2717 to 0.2824 and from -0.1376 to 0.1376 between duration of trunk rotation and PPT or NRS (Table 2).

**Table 2:** Correlation coefficient (rho-Spearman) calculated for pressure pain thresholds (PPT, kPa) and low back pain (LBP, 0–10 scale) intensity for trunk flexion and trunk rotation cut-off angles.

		PPT (kPa)		LBP intensity (0–10)	
	Angles	r	p-value	r	p-value
**Trunk forward bending**	**<30°**	0.1464	0.6024	-0.2717	0.3273
	**>30°**	-0.1464	0.6024	0.2717	0.3273
	**>60°**	-0.1571	0.5756	0.2824	0.3078
	**>90°**	0.1784	0.5247	-0.0821	0.7713
**Trunk rotation**	**<10°**	-0.1286	0.6482	-0.1376	0.6248
	**>10°**	0.1286	0.6482	0.1376	0.6248
	**>30°**	0.1321	0.6389	0.1180	0.6754

The time spent with the trunk bended forward or rotated following a median split for PPT, LBP intensity, was similar to the ones obtained for the entire population (Table 3). Furthermore, there were no significant difference between PPT values measured on the left side (PPT_left_) and the right side (PPT_right_) of the low back (Table 4).

**Table 3:** Pressure pain thresholds (PPT, kPa) and low back pain intensity (LBP, 0–10 scale) using median split and 25^th^, median 75^th^ according to cut-off angles for trunk flexion (<30°, >60°, >90°) and trunk rotation (>10°, >30°).

			PPT (kPa)			LBP intensity (0–10)		
	Angles	Median	25th	Median	75th	25th	Median	75th
**Trunk forward bending**	>30°	<69.1%	307.9	471.9	614.9	1.6	2.6	2.7
		>69.1%	224.7	294.8	453.7	2.8	3.6	5.1
	>60°	<9.2%	307.9	471.9	614.9	1.6	2.6	2.7
		>9.2%	287.6	346.7	436.7	2.8	3.6	5.1
	>90°	<0.1%	233.6	341.6	608.6	1.6	2.7	2.8
		>0.1%	287.6	346.7	453.7	2.8	3.6	5.1
**Trunk rotation**	>10°	<46.6%	181.0	280.5	469.9	2.1	3.2	4.6
		>46.6%	318.2	452.2	608.6	2.0	2.7	2.8
	>30°	<0.3%	236.8	452.2	546.5	2.0	2.6	3.0
		>0.3%	318.2	346.7	463.5	2.2	2.8	5.1

Abbreviations: PPT, pressure pain threshold; LBP, low back pain

**Table 4:** Pressure pain thresholds (kPa), 25^th^, median and 75^th^ for the 14 locations covering the low back region.

Points	25th	Median	75th
**P**_**left**_	373.4	558.0	740.3
**P**_**right**_	389.1	568.3	747.7
**P**_**all**_	381.3	563.1	744.0

#### Combined associations of the duration of forward bending and rotation of the trunk with LBP intensity or pressure pain sensitivity

**Fig 7:**
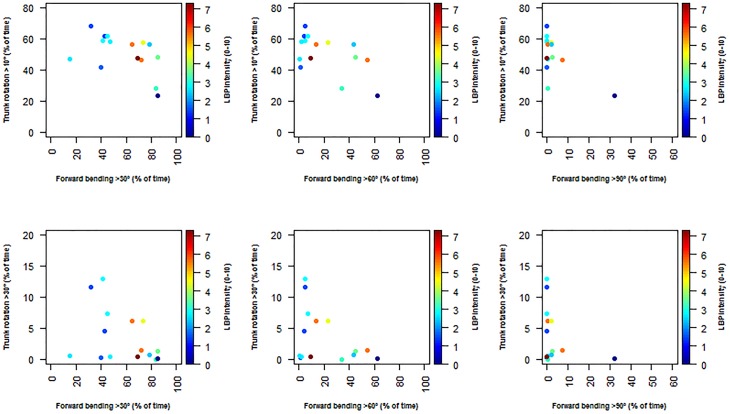
Scatter plots of the correlation between the different cut-off angles for trunk forward bending (>30°, >60°, >90°), trunk rotation (>10°, >30°) and low back pain intensity (LBP, 0–10).

**Fig 8:**
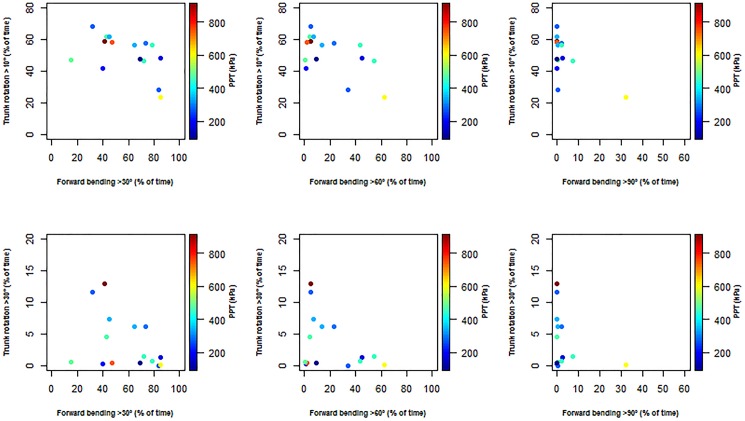
Scatter plots of the correlation between the different cut-off angles for trunk forward bending (>30°, >60°, >90°), trunk rotation (>10°, >30°) and pressure pain thresholds (PPT, kPa).

No significant association between the combined duration of forward bending and flexion of the trunk with LBP intensity or PPT was found (Figs 7 and 8).

## Discussion

Taken together, the present findings showed that vineyard-workers’ pruning activity is likely to lead to the adoption of bended and rotated postures for relatively long period of time. For instance, during the 12 minutes of pruning activity, vineyard-workers spent almost 60% of the time with the trunk bended >30°. Our results are comparable to those reported in a study specifically designed to assess the effects of different pruning trellis on the risk of WMSDs in the low back [18]. In the latter, 11 vineyard workers were asked to perform a simulated pruning task during approx. five minutes showing that vineyard-workers spend between 31% and 80% with the trunk forward bended > 30°. Once extrapolated over a working day, this result suggests that vineyard-workers spend most of their working time with trunk postures which have extensively been reported to increase the risk of LBP [15,27,28]. Interestingly, Coenen and colleagues [26] have reported that this risk is significantly amplified when the trunk is bended >60° more than 5% of the time. In our study, pruning activity largely exceeded this threshold (i.e., 21%), consequently increasing the risk of LBP among vineyard-workers. This observation is corroborated by previous studies showing that trunk forward bending negatively affects viscoelastic tissues such as ligaments, fascia, discs [44–46] and spine stability. Indeed, prolonged trunk forward bending increases the risk of ligaments laxity and ligaments micro-damages, the risk of inflammation and, consequently, the risk of LBP [44,46].

However, the Spearman rank analysis and the sensitivity analysis using a median split showed no significant relationship between the time spent in each cut-off angles for both trunk forward bending and trunk rotation with LBP intensity and pressure pain sensitivity. In other words, our results suggest no association between the duration and the angulation of trunk forward bending or trunk rotation with LBP intensity or pressure pain sensitivity. This finding is in line with recent studies questioning this relationship [16,31,32,47]. For instance, Villumsen and colleagues [32,47] have reported a negative association between the time spent with the trunk bended forward and LBP intensity in a cohort of blue-collar workers. In another study, Lagersted-Olsen and colleagues [31] questioning the relationship between the duration of forward bending and LBP over a year period have also concluded that the risk of developing or aggravating LBP is not directly associated with the duration of forward bending at work when using angles >30°, >60° and >90°.

Thus, we assess trunk rotation and we can argue that pruning activity can be considered as a task that combined trunk forward bending and trunk rotation. For instance, vineyard workers spent 50% of the 12 minutes working time with the trunk rotated >10° for pruning. Similar rotated trunk postures have been previously observed among other workers such as sheep shearers [48] or paramedics [49]. However, during the 12 minutes of pruning activity, vineyard-workers spent significantly most of the time with the trunk rotated to the left side for all cut-off angles (i.e. <10°, >10° and >30°). This result clearly suggests a trunk asymmetry between the left and right side during the performance of this task. This observation could be explained by the vineyard-workers handedness which determines whether the vineyard-worker stand on the right or left side of the vine and could explained why the pattern observed for the left-handed vineyard-worker is not different from the right-handed. Similar to longer time spent in bended postures, trunk rotation is also reported to increase lower back muscle activation and decrease ligaments laxity [50]. During a symmetric flexion task, loads are shared equitably between both sides of the spine [51,52]. However, during an asymmetric flexion task, Ning and colleagues [53] have observed on the contralateral side of the rotation an increasing tension in spine ligaments and on the ipsilateral side a longer muscle activation finally increasing the risk of LBP [26]. However, this longer muscle activation does not result in decreased PPT on the low back muscles of the ipsi or contra-lateral side of the rotation. Indeed, our results revealed no significant difference between PPT values of the left and right side of the low back confirming, for the sample size of 15 vineyard-workers, the absence of association between trunk rotation and pain sensitivity mentioned earlier.

Avoiding bended or rotated trunk postures may result in lower mechanical exposure and could consequently be considered among others as one of the main reasons given to the lack of association between high LBP intensity and time spent with the trunk forward bended or the trunk rotated [32,54]. However, in our study this explanation seems unlikely as the duration of forward bending >30° once extrapolated on a working day (i.e. almost 252 min/day) is twice higher than that reported by Villumsen and colleagues [32], i.e. 100min/day among blue-collar workers. Results of the present study could also be attributable to at least two other factors: (1) a “floor effect” as the median low back pain intensity reported by vineyard-workers is relatively low, i.e. around 3 on a 0–10 rating scale [55]; and (2) the fact that the most painful vineyard-workers may have left the profession making our vineyard-workers “healthy survivors”. This latter explanation seems particularly relevant as our sample of vineyard-workers have seniority close to 20 years. Finally, a third possible explanation recently argued by Lagersted-Olsen and colleagues [31] is that assessing separately the effect of forward bending or trunk rotation on LBP intensity can lead to miss a possible association between these outcomes. At this point and as recently suggested by Lagersted-Olsen and colleagues [31], we have assessed the combined effect of duration of forward bending and trunk rotation on LBP intensity and PPT. Our results show no significant association regarding all the possible combinations between trunk forward bending, trunk rotation cut-off angles and mean LBP intensity over the last two weeks of work or PPT. In other words, LBP intensity or pressure pain sensitivity was not affected by the combined effects of duration of forward bending and trunk rotation. However, further studies assessing this relationship among a larger sample of vineyard-workers are needed to complete our results.

This study presents several limitations. First, the rather small sample size of 15 vineyard-workers from a single castle may limit the generalizability of the results to all vineyard-workers. However, we believe that this was sufficient to generate relevant results. Indeed, it is important to mention that the number of vineyard-workers that volunteered to participate in this study represented more than 65% of the entire vineyard-workers population of the Château Larose-Trintaudon. Further, this Château is the largest vineyard in this area with almost 500 acres of vineyard and more than 1 million of bottles produced each year. Second, the method used for the kinematics analysis of vineyard-workers’ pruning activity is also not without limitations. Third, measuring trunk kinematics using a single wireless inertial measurement unit combining a 3D angular gyroscope, a 3D accelerometer and a 3D magnetometer during a fast paced activity such as pruning may have resulted in measurement error. Further, the relative short duration of the recordings (12 minutes) questions the reliability of the data. Indeed, previous studies have assessed physical exposure at work over an entire or several working days [26–28,46,56,57]. At this point, however, it is conceivable that the nature of the professional task (e.g., variety, repetitiveness…) is an important factor that should influence the appropriate duration and frequency of recordings. Hence, unlike the above mentioned studies assessing a wide range of physical exposure among numerous working sectors such as metal, chemical, food and wood sectors [26–28,56], pruning task is considered highly repetitive and rather monotonous [18,20]. That is the reason why we are confident to consider a 12 minutes recording as sufficient to compute reliable kinematic data and to obtain a realistic picture of the adopted postures during pruning. Of note, Kato and colleagues [18] have asked 11 vineyard-workers to perform pruning during 5 minutes to assess the effects of different pruning trellis on trunk postures, whereas Roquelaure and colleagues [20] have analyzed pruning activity of six vineyard-workers for approximately 8 minutes to conclude that pruning activity lead to the adoption of extreme wrist postures. Fifth, it is noteworthy that the presence of examiners during the performance of pruning activity may have changed vineyard-workers working habits. In this sense, the exposure to bended or rotated postures should have been underestimated [48]. After all and even if PPT measurements do present advantages like the link with musculoskeletal pain and its semi-objective character [58–60], PPT cannot be considered as a substitution tool for objective diagnoses of LBP. However, the sensitivity analysis performed in this study and the high percentage of non-specific LBP reported among the entire population (i.e. almost 90%) [61] lead us thinking that our results were not affected by the absence of objective diagnosis. Despite these limitations, the present study assessing vineyard-workers activities is the necessary first step before developing and implementing adapted interventions [62]. Still prospective studies are needed to determine the effects of work exposure on LBP. Finally, we have also conducted analyses to assess the effect potential well known LBP confounders such as gender, age, weight and BMI [9,63] on trunk kinematics and risk of LBP. Although our analyses revealed that women spent significantly more time with the trunk flexed >60° and that age, weight and BMI did not change LBP intensity and PPT values, our small sample size prevents us from being able to generalize our findings.

## Conclusions

This field study revealed that vineyard-workers adopt forward bended and rotated trunk postures that may increase the risk of WMSDs in the low back during the execution of pruning activity. Indeed, more than half of the assessed working time was spent with the trunk flexed greater than 30° and more than 20% with the trunk rotated greater than 10°. Then, our study has also pointed out a significant difference between left and right rotation of the trunk. However, our study did not reveal any relationship between duration of forward bending or trunk rotation and LBP intensity or pressure pain sensitivity. Finally, this study reinforces the necessity of further field measurements with longer time of observation and larger sample size to confirm our findings and to investigate other variables specifically the effects of potential LBP confounders such as gender, age or job seniority to accurately quantify the risk exposure.

## Abbreviations

BMI: Body mass index
LBP: Low back pain
PPT: Pressure pain thresholds
WMSDs: Work related musculoskeletal disorders
